# Perceived levels of social stigma following HIV notification: Insights from Brazilian blood centers

**DOI:** 10.1016/j.bjid.2024.104480

**Published:** 2024-11-15

**Authors:** Renata Buccheri, Carolina Miranda, Cesar de Almeida-Neto, Thelma Gonçalez, Liliana Preiss, Luiz Amorim, Anna Barbara Carneiro-Proiett, Paula Loureiro, Ester C. Sabino, Brian Custer

**Affiliations:** a Vitalant Research Institute, San Francisco, USA; b Fundação Hemominas, Belo Horizonte, MG, Brazil; c Fundação Pro-Sangue Hemocentro de São Paulo, São Paulo, SP, Brazil; d Research Triangle Institute (RTI) International, Rockville, USA; e Fundação Hemorio, Rio de Janeiro, RJ, Brazil; f Fundação Hemope, Recife, PE, Brazil; g Faculdade de Medicina da Universidade de Pernambuco, Recife, PE, Brazil; h Faculdade de Medicina da Universidade de São Paulo, São Paulo, SP, Brazil; i University of California San Francisco, San Francisco, USA

**Keywords:** Hiv-related stigma, Hiv/aids, Blood donors, Brazil

## Abstract

**Background:**

HIV/AIDS remains a highly stigmatizing disease worldwide, preventing people with risk or infection from testing to learn their HIV status, accessing supportive services, or taking antiretroviral therapy. Despite many studies of HIV in blood donors, no studies have evaluated the factors that contribute to stigma surrounding this illness following notification process and counseling provided by blood centers.

**Methods:**

A cross-sectional questionnaire-based survey was conducted between 2016 and 2017. Persons with HIV were invited to return to the blood center for an audio computer-assisted interview after participation in an HIV risk factor assessment study conducted from 2007 to 2016. The questionnaire was based on HIV risk interviews developed by the US CDC, with modifications appropriate to the Brazilian setting which aimed to evaluate their follow-up activities, perceptions of HIV stigma and discrimination, and the quality of counseling and notification after the donation that tested positive for HIV. Response frequencies and adjusted odds ratios from multivariable logistic regression analyses are reported.

**Results:**

268 HIV-positive blood donors agreed to participate in the study. Almost all participants, 262 (97 %), rated as very important or important the blood center counseling experience in their decision to seek health care. One-hundred-five (39 %) participants reported none to low levels of stigma, and 163 (61 %) participants moderate stigma. Individuals reporting heterosexual orientation (OR=2.13, 95 % CI [1.08‒4.22]) and healthcare-seeking behavior (OR=2.46, 95 % CI [1.10‒5.48]) had significantly increased odds of reporting moderate levels of stigma.

**Conclusions:**

Our study provides information about perceived stigma and discrimination in the Brazilian blood donor population and reinforces the importance of the counseling process in linkage to care and reducing HIV-related stigma.

## Introduction

HIV prevention and treatment therapies have had major advancements in the last 20 years; however, HIV remains a highly stigmatized disease worldwide, preventing people with risk or infection from seeking prevention tools, learning their HIV status, accessing support services, or adhering to antiretroviral therapy.^1^^,^^2^ The concept of stigma is complex, and how stigma manifests may vary among different cultures and groups of people, but its impact on HIV-related health outcomes is consistently detrimental.^3^

Perception of stigma among those with HIV has been correlated with a late presentation for treatment.^4^ Despite many studies on HIV in blood donors, to date, no studies have evaluated the linkage with health care services after the HIV notification counseling conducted by the blood bank nor how the stigma surrounding this illness affects this specific group of individuals.

Our study aimed to assess whether HIV-positive donors who returned to the blood center for notification later attended HIV counseling referral centers for additional treatment and follow-up and investigate factors associated with HIV-positive donors' perceived levels of social stigma.

## Materials and methods

### Study design and population

The REDS-III Brazil notification and linkage to care study was a cross-sectional questionnaire-based survey conducted in four large blood centers, Fundação Pro-Sangue (São Paulo), Hemominas (Belo Horizonte), Hemope (Recife), and Hemorio (Rio de Janeiro). The study population was a consecutive convenience sample of 18‒69-year-old blood donors who tested HIV-positive, and who had previously participated in a study to evaluate risk factors for HIV infection conducted during the period of 2007–2016. Participants 18-year of age or older were contacted by letter or telephone and invited to return to the blood center to participate in this study and complete an Audio Computer-Assisted Structured Interview (ACASI) to evaluate their follow-up activities regarding HIV infection status and treatment, HIV stigma and discrimination, and quality of the counseling and notification by the blood center. A single study visits to the blood center occurred between February 2016 to June 2017. A $US 35 (75 Reais) participation reimbursement was provided to each person who completed the ACASI. To assess evidence of linkage to care, we collaborated with Brazil Ministry of Health to obtain data on HIV treatment and disease progression monitoring. The Brazilian National HIV treatment and progression databases of the Ministry of Health (MoH) contain information such as viral load and CD4 and CD8 T-lymphocyte count, indicating the use of health services to monitor HIV infection.

### Ethical considerations

This study was approved by the Brazilian National Ethical Committee, local ethical committees at each participating center, the Institutional Review Board (IRB) of the University of California San Francisco in the United States, the IRB of record for Vitalant Research Institute, and the Research Triangle International IRB. Research staff ensured that all participants provided written informed consent before participating in the study. This consent included seeking approval to access participant's HIV monitoring data from Brazil Ministry of Health.

### ACASI interview procedure

Each participant completed a self-administered survey in ACASI format (NOVA Research Company, Questionnaire Development System v3.0, Silver Spring, Maryland) of approximately 30 min in length in a private location. The ACASI included content on demographic characteristics, including age, race, employment status, education level, marital status, sexual orientation, and blood donation history (first-time or repeat donor) at the time of their HIV positive donation. Participants were asked about initial linkage to HIV care following notification of HIV-infection status by the blood center and whether they were currently taking ARV medications at the time of the study visit. Participants also reported awareness of HIV status before donation and attempt to donate blood after HIV notification.

Assessment of HIV-related stigma and discrimination was measured by a group of 26 questions on disclosing HIV status and to whom this information was revealed, reasons for not disclosing HIV status, if respondents ever felt discriminated against, and experiences of situations in which they perceived discrimination. Participants were also asked to rate the HIV notification process by the blood center. The questionnaire was based on the HIV risk interviews developed by the US CDC with modifications appropriate to the Brazilian setting.^5^^,^^6^

### Linkage to ministry of health hiv information

The Brazilian National HIV treatment and disease progression database was created in 2002 by the MoH. Each Brazilian Voluntary Counseling Center (VCT) organization reports information on individuals with HIV/AIDS (viral load, CD4, and CD8 information) to a central data coordinating office in the capital of Brazil, Brasília. This information is essential for health services planning and strategies, focusing on affected individuals and communities to provide indicators of success and to support efforts to manage HIV in Brazil. Participants' disease monitoring information was obtained from the Brasilia Information System of Testing and Counseling Centers (SI-CTA) laboratory data for each HIV-positive blood donor based on identifiers the REDS-III study team securely transmitted to the MoH. The data were merged by Brasilia SI-CTA staff, and those records for study participants who successfully linked to MoH data and had consented were returned to the study.

### Data analysis

A dataset for each blood center site containing ACASI questionnaires information and the Brazil MoH monitoring data such as CD4, CD8, and viral load were extracted and transferred to RTI by secure methods and merged with the donor and donation information to create a dataset with notification status data and donor demographics. Data from different datasets from the same blood center were merged using the blood bank registration ID number (donor ID) and donation date. All data were captured in statistical analysis programs for analysis. Datasets were created and cleaned using SAS v7 (SAS Institute, Cary, North Carolina). We conducted data analysis with Stata version 16.1 (StataCorp, College Station, Texas). For all inferential statistics, significance was defined as *p* < 0.05.

Descriptive statistics and frequencies were calculated for demographic characteristics and survey responses. A scale was constructed to measure the presence of stigma and discrimination by assigning one point for each answer to the 26 questions that evaluated discrimination or perceived self-stigma (Supplemental Material). The distribution of raw scores was displayed in a histogram and later categorized for analytical purposes. Participants with a score below six were classified as having none to low levels of stigma (reference category), those with a final score equal or above six as having moderate stigma, and equal and above 13 as having a high stigma level. This categorization was performed after data collection.

The relationship of three levels of self-reported stigma to various demographic and survey responses was explored using Chi-Square tests. Logistic regression was used to assess the effect of predictors where there was statistical evidence of an association of self-reported stigma. We built bivariate models and a final multivariable model by a forward selection approach, including those variables with bivariate p-values <0.1. We report Odds Ratios (OR), 95 % Confidence Intervals, and p-values, for which we assumed two-sided distributions and an alpha error probability of 0.05.

## Results

The potential study population consisted of 720 confirmed HIV-positive blood donors who had participated in the previous HIV risk factor study. Of these, 268 (37 %) agreed to participate in the current protocol and were included in this study. The median time elapsed between the HIV-positive blood donation date to the completion of the ACASI interview was of 5 years (range, 2‒17). Table 1 provides the donors' demographic variables and donor status.

**Table 1:** Baseline social-demographic characteristics of blood donors notified with HIV in four blood centers in Brazil.

	HIV-positive blood donors included in the study (*n* = 268)	HIV-positive blood donors not in study (*n* = 452)
**Variables**	**n (%)**	**n (%)**
**Gender**		
Male	207 (77.2)	363 (80.3)
Female	61 (22.7)	89 (19.6)
**Age group**^a^		
18‒25	16 (6.0)	106 (23.5)
26‒30	52 (19.7)	93 (20.6)
31‒39	93 (35.3)	136 (30.2)
40+	103 (39.0)	116 (25.7)
**Self-reported race**^b^		
White	96 (36.3)	175 (38.7)
Black/Mixed (“Pardo”)	136 (51.5)	229 (50.6)
Asian/Native/Other	31 (11.7)	47 (10.4)
Unknown/Refused	1 (0.3)	1 (0.2)
**Blood Center**		
HEMOPE ‒ Recife	99 (36.9)	166 (36.7)
FPS ‒ São Paulo	75 (27.9)	79 (17.4)
HEMOMINAS ‒ Belo Horizonte	37 (13.8)	63 (13.9)
HEMORIO ‒ Rio de Janeiro	57 (21.2)	144 (31.8)
**Donor status**^c^^,^^d^		
First time	119 (46.3)	214 (50.8)
Repeat	138 (53.7)	207 (49.1)
**Marital Status**		
Single	126 (47.0)	237 (52.4)
Living together, married	105 (39.1)	168 (37.1)
Divorced	30 (11.1)	38 (8.4)
Widowed	7 (2.6)	9 (1.9)
**Sexual orientation**^e^		
Heterosexual	150 (55.9)	264 (58.5)
Bisexual	36 (13.4)	74 (16.4)
Homosexual	70 (26.12)	99 (21.9)
Unknown/Refused	12 (4.48)	14 (3.1)
**Education**		
Never been to school	‒	11 (2.4)
Elementary School	65 (24.2)	2 (0.4)
High School	89 (33.2)	50 (11.0)
Technical or Professional School	27 (10.0)	204 (45.7)
University/Pos-graduation	87 (32.4)	184 (40.7)
Unknown/Refused	‒	1 (0.2)
**Employment status**		
No	85 (31.7)	‒
Yes	183 (68.2)	‒

a Data were missing for four participants in the study population group and 1 participant in the blood donors’ group.

b Data were missing for four participants in the study population group.

c Data were missing for eleven participants in the study population group.

d Data were missing for two participants in the study population group.

e Data were missing for five participants in the blood donors group.

When asked if participants knew about their HIV infection before donation, 13 out of 263 (5 %) answered that they were aware of their status before donation, and 4 (31 %) said they tried to donate blood again. Among the 249 participants unaware of their HIV infection before donation, 7 (3 %) tried to donate blood after the blood center HIV notification and counseling. Two hundred thirty-three participants (89 %) reported seeking medical care after HIV notification and counseling from the blood center, and 140 (60 %) of these sought medical care within two weeks of blood center notification (Tables 2 *and* 3*). T*wo hundred twenty-three *(83 %) participants reported* taking ARV medications at the time of their study visit.

**Table 2:** HIV-positive blood donor linkage to care.

After blood center notification, where did you go to seek health care?	*n* = 233	%
Volunteer Counseling and Testing site	64	28
Private Hospital	21	9
Public Hospital	126	54
My Physician's Office	14	6
Other	8	3
**After blood center notification, how long did it take for you to seek health care?**	***n* = 233**	**%**
Within 2-weeks	140	60
2‒4 weeks	44	19
1‒3 months	19	8
4‒6 months	7	3
>6-months	20	9
Unknown/Refused	3	1

**Table 3:** First record of CD4 and viral load after the HIV-positive donation in the Brazil Ministry of Health HIV disease monitoring program.

	n	Median	IQR	Range
**CD4 Lymphocyte Count (cell/mm^3^)**	228	470	282‒681	1‒1425
**HIV Viral Load (copies/mL)**	113^a^	9721	3375‒34,136	77‒2857.276

a After donation, 113 out of 122 individuals had viral loads available in their first record at the MoH.

Two hundred and sixty-two participants (99.6 %) consented to allow access to their HIV disease monitoring data in the Brazilian MoH database. However, Brasilia SI-CTA could only link data for 229 (87 %) of the participants. Of the 229, at some point after the blood center HIV notification, 107 individuals had CD4 data, 121 had CD4 and viral load data, and one participant had only viral load data in the system.

When the ACASI participants were asked to rate the HIV notification and counseling performed by the blood center, 231 (86 %) answered that the counseling skills of the blood center physician during their HIV notification were very satisfactory or satisfactory. When asked to rate their confidence in the blood center physician's approach to notification, 246 (92 %) responded that they were very confident or confident. Almost all participants, 262 (97 %), rated as very important or important the blood center counseling in their decision to seek health care.

Using the calculated stigma index, 105 (39 %) participants were classified as having none to low levels of stigma, and 163 (61 %) participants with moderate stigma. No one reported high levels of stigma. No differences in stigma levels were observed between the groups regarding age group, gender, self-reported race, marital status, and the blood center where participants were notified of their HIV infection.

We conducted a bivariate analysis to examine the association between demographic variables and perceived levels of stigma. Our findings revealed that individuals reporting heterosexual orientation were more likely to self-report moderate stigma compared to those reporting homosexual orientation (OR = 2.19, 95 % CI [1.21‒3.94]). However, no differences in stigma levels were observed between individuals reporting bisexual or homosexual orientation (OR = 0.95, 95 % CI [0.40‒2.24]). Moderate stigma level was more likely for individuals who reported not being employed (OR = 1.69, 95 % CI [1.01‒2.85]), and for those who reported elementary school education level (OR = 3.77, 95 % CI [1.40‒10.16]), compared to those with technical or professional school.

Knowing someone with HIV infection, currently taking antiretroviral therapy, and having heard of or previously tested in a counseling and testing center showed no association with self-reported stigma level. There was a significant difference in the odds of reporting moderate levels of stigma among individuals who sought health care after HIV status notification by the blood center compared to those that did not seek health care (OR = 2.75, 95 % CI [1.04‒4.94]).

Sexual orientation, education level, employment status, and healthcare-seeking behavior were selected for the final model logistic regression. Adjusting for other predictors, we found that seeking health care significantly increased the odds of self-reported moderate stigma level (OR=2.46, 95 % CI [1.10‒5.48]) compared to the reference level. Respondents who reported heterosexual orientation had significantly increased odds of reporting moderate levels of stigma compared to those who reported homosexual orientation (OR = 2.13, 95 % CI [1.08‒4.22]) (Table 4).

**Table 4:** Unadjusted and adjusted logistic regression evaluating factors associated with perceived levels of stigma.

Characteristics	Unadjusted		Adjusted Model	
	Odds Ratio	p-value	Odds Ratio^a^	p-value
	(95 % CI)		(95 % CI)	
**Sexual orientation**				
Homosexual	Ref.		Ref.	
Bisexual	0.95 (0.40‒2.24)	0.92	0.97 (0.40‒2.32)	0.95
Heterosexual	2.19 (1.21‒3.94)	0.009	2.13 (1.08‒4.22)	0.029
**Employment status**				
Yes	Ref.		Ref.	
No	1.69 (1.01‒2.85)	0.045	1.48 (0.84‒2.60)	0.17
**Education level**				
Technical or Professional School	Ref.		Ref.	
University/Pos-graduation	2.43 (0.93‒6.34)	0.069	2.55 (0.95‒6.82)	0.06
High School	2.03 (0.77‒5.30)	0.147	1.66 (0.61‒4.49)	0.31
Elementary School	3.77 (1.40‒10.16)	0.009	2.35 (0.80‒6.86)	0.11
**HIV Healthcare Seeking Behavior**				
No	Ref.		Ref.	
Yes	2.75 (1.04‒4.94)	0.038	2.46 (1.10‒5.48)	0.027

a All variables adjusted for all other variables in column.

## Discussion

The relationship between HIV infection, stigma, and accessing health care services is complex and likely varies between populations. Our study examined the impact of perceived HIV-related stigma on blood donors who were notified about their HIV status through blood donation. Among donors with HIV from four different regions of Brazil, more than half reported experiencing moderate HIV stigma and discrimination. We found that the odds of experiencing perceived levels of moderate stigma and discrimination were over two times higher among respondents who sought health care. Reporting heterosexual orientation was strongly associated with experiencing HIV stigma compared to those reporting homosexual orientation. Based on other research, it has been observed that the magnitude of stigma can vary and appears to be higher for people living with HIV who have not yet disclosed their serostatus.^7^^,^^8^ Although in our study stigma was present, its intensity was not a major barrier in this population of blood donors since the vast majority of the respondents reported having disclosed their serological status to others.

Furthermore, black/mixed race, lower education, residing in a less developed region, and high levels of social vulnerability are all associated with a higher likelihood of presenting to care with more advanced diseases, not using antiretroviral therapy, and not achieving viral suppression in Brazil and in other countries.^9^, ^10^, ^11^, ^12^ Intersectional stigma is the convergence of belonging to multiple stigmatized groups (e.g., HIV status, sexual orientation, race, socio-economic status) and may intensify negative detrimental health effects.^13^^,^^14^ However, contrary to our expectations, we found that stigma was not strongly associated with self-reported black/mixed race or gender in our study population. Even though the group of people with lower educational attainment showed a higher level of stigma, this finding was not significant in multivariable analysis. On the other hand, those who declared heterosexual orientation experienced significantly higher levels of HIV-related stigma previously described as being related to the acceptance of their status, which may be influenced by their negative perception of HIV, conceptions of masculinity, and their fear of experiencing homophobia.^15^, ^16^, ^17^

Prior research identified HIV-related stigma and discrimination as associated with lower levels of HIV treatment engagement,^18^^,^^19^ healthcare-seeking behavior,^7^^,^^20^ and access to HIV care.^2^ In contrast, our data indicate that among Brazilian blood donors, there is a significant positive association between the likelihood of seeking health care services and the presence of moderate self-reported stigma. Most study participants were on antiretroviral therapy. This relationship may have been found because our participants were a convenience sample willing to participate in the study and may have been more likely to engage with health care services. In addition, almost all individuals reported blood bank counseling as important or very important in their decision to seek health care. Most likely, the impact of a good counseling process was more meaningful for those participants who reported a higher level of stigma and experienced more discrimination. Increasing disease awareness among those who feel marginalized due to a specific condition is critical to undoing misconceptions or beliefs regarding HIV/AIDS and can encourage coping strategies, of which the decision to seek care is a key part.

There are limitations in our study. A validated stigma questionnaire to measure HIV-related stigma in the Brazilian setting was not available, and we may have missed important components of measurement of stigma and discrimination in Brazilian culture. The time elapsed between the HIV-positive donation and the return to the blood center to complete the questionnaire was varied, with a median of 5 years. This long interval between blood donation and participation in the study may have partially underestimated the number of individuals who reported stigma since stigma can attenuate over time, especially in those diagnosed more than five years ago.^2^ We also recognize the size of the group participating in the study may limit the generalizability of the findings, even to all donors with HIV who are notified of their serostatus by blood centers in Brazil. Finally, our findings are not generalizable to all blood donors with infection. Differences are evident among those who did and did not participate. For example, we had less success enrolling those who were younger and had lower educational attainment at the time of the HIV-positive donation. Additionally, the participants in the study are likely the most engaged, as they were drawn from the pool of previous blood donors we successfully contacted, and they voluntarily participated in this study. Those previous donors we couldn't contact or who declined to participate may exhibit differing levels of perceived stigma and access to health services.

## Conclusion

Our study provides previously unreported information about stigma and discrimination in the Brazilian blood donor population, highlighting the importance of blood center counseling in facilitating linkage to care and reducing stigma among individuals recently diagnosed with HIV. The findings suggest that heterosexual orientation and healthcare-seeking behavior are associated with higher levels of perceived stigma, emphasizing the need for targeted interventions to address HIV-related stigma in these groups.

## Author's contribution

All authors contributed to the article and approved the submitted version.

## Conflicts of interest

The authors declare no conflicts of interest.

## Appendix Supplementary materials

Image, application 1
