# Supplementary material for: Perceived levels of social stigma following HIV notification: Insights from Brazilian blood centers
Source: Braz J Infect Dis. 2024 Nov 15;28(6):104480. doi: 10.1016/j.bjid.2024.104480 (PMC11609526; doi:10.1016/j.bjid.2024.104480)
Supplement: Supplementary file 1 [file mmc1.docx]

**BJID-D-24-00154_Supplementary Material**

**Supplemental Material**

| **Twenty-six questions and responses related to self-stigma and discrimination** | | **Yes** | **%** | **n** |
| --- | --- | --- | --- | --- |
| **1** | **After you learned about your HIV test result, have you disclosed your result to someone else?** | 197 | 73 | 268 |
| **2** | **To whom have you disclosed your HIV test result?** |  |  | 197 |
| 2a | My partner/spouse | 116 | 59 |  |
| 2b | Best friend | 76 | 39 |  |
| 2c | Several friends | 21 | 11 |  |
| 2d | Family | 91 | 46 |  |
| 2e | Co-worker(s) | 15 | 8 |  |
| 2f | Priest/Religious | 17 | 9 |  |
| 2g | Health care provider | 43 | 22 |  |
| 2h | Other | 4 | 2 |  |
| **3** | **What are the reasons for not disclosing your HIV test result?** |  |  | 71 |
| 3a | I'm afraid of discrimination. | 37 | 52 |  |
| 3b | I feel ashamed/embarrassed. | 26 | 37 |  |
| 3c | I believe this is my personal/private information. | 43 | 61 |  |
| 3d | Other reason. | 9 | 13 |  |
| **4** | **Have you felt discriminated against because of your HIV test result?** | 124 | 44 | 268 |
| **5** | **Did something occur that you felt was discrimination?** |  |  | 124 |
| 5a | You have been discriminated against by coworkers or classmates because of your HIV test result. | 11 | 9 |  |
| 5b | Someone has made fun of you because of your HIV test result. | 18 | 14 |  |
| 5c | Someone has offended you because of your HIV test result. | 15 | 12 |  |
| 5d | You have been harassed because of your HIV test result. | 7 | 6 |  |
| 5e | All above | 4 | 3 |  |
| 5f | Other | 76 | 6 |  |
| **6** | **Do you feel that people avoid you because of your HIV test result?** | 16 | 13 | 268 |
| **7** | **Has anyone forced you to move out of a place you lived because of your HIV test result?** | 6 | 2 | 268 |
| **8** | **Have you been refused housing because people suspect you might have HIV?** | 2 | 0 | 268 |
| **9** | **Has a health care provider made you feel bad because of your HIV test result?** | 57 | 21 | 268 |
| **10** | **Has a health care provider refused to touch you because of your HIV test result?** | 18 | 7 | 268 |
| **11** | **Have you been refused medical care or denied hospital services because of your HIV test result?** | 9 | 3 | 268 |
